# Interval-Valued Optimization Problems Involving (*α*, *ρ*)-Right Upper-Dini-Derivative Functions

**DOI:** 10.1155/2014/750910

**Published:** 2014-03-27

**Authors:** Vasile Preda

**Affiliations:** ^1^Faculty of Mathematics and Computer Science, University of Bucharest, 010014 Bucharest, Romania; ^2^Institute of Mathematical Statistics and Applied Mathematics of the Romanian Academy, 050711 Bucharest, Romania; ^3^National Institute of Economic Research, 050711 Bucharest, Romania

## Abstract

We consider an interval-valued multiobjective problem. Some necessary and sufficient optimality conditions for weak efficient solutions are established under new generalized convexities with the tool-right upper-Dini-derivative, which is an extension of directional derivative. Also some duality results are proved for Wolfe and Mond-Weir duals.

## 1. Introduction

Recently, Yuan and Liu [1] considered some new generalized convexity concepts using right upper-Dini-derivative, which is an extension of directional derivative. Thus some optimality and duality results are established for a nondifferentiable multiobjective programming problem. For various approaches relative to generalized convexity, we refer to [2–9].

In many real-life situations data suffer from inexactness. The interval-valued optimization problems are closely related to optimization problems with inexact data. Recently, Wu [10–12] derived optimality conditions and duality results for a multiobjective programming problem with interval-valued objective functions. See also [13] and their references.

In this paper we consider an interval multiobjective optimization problem. Some new optimality conditions and duality results are stated under new generalized convexities with the tool-right upper-Dini-derivative. The paper is organized as follows. In Section 2 some definitions, notations, and some basic arithmetic of interval calculus are given. In Section 3, we state necessary optimality conditions and in Section 4 we present sufficient optimality conditions. The duality results are stated in Sections 5 and 6. The last section gives some conclusions.

## 2. Notations and Preliminaries

Let ℝ^*n*^ be the *n*-dimensional Euclidean space and let ℝ_+_^*n*^ be its nonnegative orthant. For *x* = (*x*_1_,…, *x*_*n*_) ∈ ℝ^*n*^ and *y* = (*y*_1_,…, *y*_*n*_) ∈ ℝ^*n*^ we consider the following conventions:

(1) $$\begin{array}{c} x≦y\;\text{iff}\;x_{i}{≦y}_{i},\;i=\bar{1,n}\;\left(\text{i}.\text{e}.,\;y_{i}-x_{i}\in {\mathbb{R}}_{+},i=\bar{1,n}\right);\\ x\leq y\;\text{iff}\;x≦y,\;x\neq y;\\ x<y\;\text{iff}\;x_{i}<y_{i},\;i=\bar{1,n}. \end{array}$$

Let *X* ⊂ ℝ^*n*^ be an arcwise connected set in Avriel and Zang [14] and Bhatia and Mehra [15] and *φ* a real-valued function defined on *X*. Let *x*_1_, *x*_2_ ∈ *X*, and *H*_*x*_1_,*x*_2__ be the arc connecting *x*_1_ and *x*_2_ in *X*.

**Definition 1**

The right derivative (or right differential) of *φ* with respect to *H* at *t* = 0 is defined as

(2) $$\begin{array}{c} \varphi^{\prime }\left(H_{x_{1},x_{2}}\left(0^{+}\right)\right)=\underset{t\to 0^{+}}{\lim}\frac{\varphi \left(H_{x_{1},x_{2}}\left(t\right)\right)-\varphi \left(x_{1}\right)}{t}. \end{array}$$

Yuan and Liu [1] give some new generalized convexity with the upper-Dini-derivative concept.

**Definition 2**

The right upper-Dini-derivative relative to *H* is defined by

(3) $$\begin{array}{c} {\left(d\varphi \right)}^{+}\left(H_{x_{1},x_{2}}\left(0^{+}\right)\right)=\underset{t\to 0^{+}}{\lim\sup}\frac{\varphi \left(H_{x_{1},x_{2}}\left(t\right)\right)-\varphi \left(x_{1}\right)}{t}. \end{array}$$

**Definition 3**

*X* is locally arcwise connected (*LAC*) at $\bar{x}$ if for any *x* ∈ *X* and $x\neq \bar{x}$ there exists a positive number $a(x,\bar{x})$, with $0<a(x,\bar{x})≦1$ and a continuous arc $H_{\bar{x},x}$ s.t. $H_{\bar{x},x}(t)\in X$ for any $t\in \left(0,a(x,\bar{x})\right)$. The set *X* is *LAC* if *X* is *LAC* at any *x* ∈ *X*.

**Definition 4 (see [1])**

Let *X* ⊂ ℝ^*n*^ be a LAC set and let *φ* : *X* → ℝ be a real function defined on *X*. The function *φ* is said to be (*α*, *ρ*)-right upper-Dini-derivative locally arcwise connected with respect to *H* at *u*, if there exist real functions *α* : *X* × *X*→ℝ and *ρ* : *X* × *X*→ℝ such that

(4) φ(x)−φ(u)≧α(x,u)(dφ)+(Hu,x(0+))+ρ(x,u),∀x∈X.

If *φ* is (*α*, *ρ*)-right upper-Dini-derivative locally arcwise connected (with respect to *H*) at *u* for any *u* ∈ *X*, then *φ* is called (*α*, *ρ*)-right upper-Dini-derivative locally arcwise connected (with respect to *H*) on *X*.

**Definition 5 (see [1])**

A *k*-dimensional vector-valued function *f* : *X* → ℝ^*k*^ is called (*α*, *ρ*)-right upper-Dini-derivative arcwise connected (with respect to *H*) at *u*, if the *i*th component of *f* is (*α*_*i*_, *ρ*_*i*_)-right upper-Dini-derivative arcwise connected (with respect to *H*) at *u* for *i* ∈ *K*, where *α* = (*α*_1_,…,*α*_*k*_)^*T*^  and  *ρ* = (*ρ*_1_,…,*ρ*_*k*_)^*T*^. If *f* is (*α*, *ρ*)-right upper-Dini-derivative arcwise connected (with respect to *H*) at any *u* ∈ *X*, then *f* is called (*α*, *ρ*)-right upper-Dini-derivative arcwise connected (with respect to *H*) on *X*.

**Definition 6 (see [3])**

A *k*-dimensional vector-valued function *f* : *X* → ℝ^*k*^ is called convex-like (with respect to *η*) on *X* if for all *x*, *u* ∈ *X* and any *t* ∈ [0,1], there exists *z* ∈ *X* such that *f*(*z*)≦*tf*(*x*)+(1 − *t*)*f*(*u*).

**Definition 7 (see [1])**

A *k*-dimensional vector-valued function *f* : *X* → ℝ^*k*^ is called *ρ*-generalized (strong) pseudoright upper-Dini-derivative arcwise connected (with respect to *H*) at *u*, if there exists vector-valued function *ρ* such that *f*(*x*)<(≦)*f*(*u*)⇒(*dφ*)^+^(*H*_*u*,*x*_(0^+^)) < *ρ*(*x*, *u*), for *x* ∈ *X*; *f* is called *ρ*-generalized (weak) quasi-right upper-Dini-derivative arcwise connected (with respect to *H*) at *u*, if there exists vector-valued function *ρ* such that *f*(*x*)≦(<)*f*(*u*)⇒(*dφ*)^+^(*H*_*u*,*x*_(0^+^))≦*ρ*(*x*, *u*), for *x* ∈ *X*, where *ρ* = (*ρ*_1_,…, *ρ*_*k*_)^*T*^.

**Lemma 8 (see [16])**

Let *S* ⊂ ℝ^*n*^ be a nonempty set and let Ψ : *S* → ℝ^*m*^ be a convex-like vector-valued function on *S*. Then either Ψ(*x*) < 0 has a solution *x* ∈ *S*, or there exists *λ* ∈ ℝ_+_^*m*^ such that the system *λ* ^*T*^Ψ(*x*)≧0 holds for all *x* ∈ *S*, but both are never true at the same time.

Let CBI(ℝ) be the class of all closed and bounded intervals in ℝ. Thus if *a* = [*a* ^*L*^, *a* ^*U*^]∈ CBI(ℝ), we have

(5) $$\begin{array}{c} a=\left[a^{L},a^{U}\right]=\left\{x\in \mathbb{R}:a^{L}≦x{≦a}^{U}\right\}, \end{array}$$

where   *a* ^*L*^ and *a* ^*U*^ mean lower and upper bounds of *a*. If *a* ^*L*^ = *a* ^*U*^, then *a* = [*a*, *a*] is a real number. Also, let *b* = [*b* ^*L*^, *b* ^*U*^]. Then, by definition we have

(6) $$\begin{array}{c} a+b=\left[a^{L}+b^{L},a^{U}+b^{U}\right],\\ a-b=\left[a^{L}-b^{U},a^{U}-b^{L}\right]. \end{array}$$

For a real number *α*, we have

(7) $$\begin{array}{c} \alpha a=\{\begin{array}{cc} \left[\alpha a^{L},\alpha a^{U}\right], & \text{if}\;\alpha >0,\;\\ \left[\alpha a^{U},\alpha a^{L}\right], & \text{if}\;\alpha <0,\;\\ \left[0,0\right]=0, & \text{if}\;\alpha =0. \end{array} \end{array}$$

Using [17, 18], we consider some preliminary results about interval arithmetic calculus.

**Definition 9**

Let *a* = [*a* ^*L*^, *a* ^*U*^]  and *b* = [*b* ^*L*^, *b* ^*U*^]∈ CBI(ℝ). We say that *a* is less than *b* and write *a*≺*b* if *a*_*i*_≺*b*_*i*_, $\forall i=\bar{1,n}$.

**Definition 10**

Let *a*, *b*∈ CBI(ℝ^*n*^). We say that *a* is less than or equal to *b* and write *a*≼*b* if *a* ^*L*^≦*b* ^*L*^ and *a* ^*U*^≦*b* ^*U*^.

Let *X* be a nonempty subset of ℝ^*n*^. A function *φ* : *X*→CBI(ℝ) is called an interval-valued function. In this case,

(8) $$\begin{array}{c} \varphi \left(x\right)=\left[\varphi^{L}\left(x\right),\varphi^{U}\left(x\right)\right], \end{array}$$

with *φ* ^*L*^, *φ* ^*U*^ : *X*⟶ℝ, *φ* ^*L*^(*x*)≦*φ* ^*U*^(*x*), ∀*x* ∈ *X*.

We consider the following multiobjective interval-valued optimization problem:

(IVP)cmin  f(x)=(f1(x),…,fn(x))subject  to  g(x)≦0    x∈X,

with *g* = (*g*_1_,…,*g*_*m*_)^*T*^, *f* ^*L*^ = (*f*_1_^*L*^,…,*f*_*k*_^*L*^)^*T*^, *f* ^*U*^ = (*f*_1_^*U*^,…,*f*_*k*_^*U*^)^*T*^, where *f*_*i*_^*L*^, *f*_*i*_^*U*^ : ℝ^*n*^ → ℝ, *f*_*i*_(*x*) = [*f*_*i*_^*L*^(*x*), *f*_*i*_^*U*^(*x*)] for $i=\bar{1,k}$ and *g*_*j*_ : ℝ^*n*^ → ℝ, $j=\bar{1,m}$. Let *X*_0_ = {*x* ∈ *X* : *g*(*x*)≦0} be the set of all feasible points of ((IVP)). We put *K* = {1,…, *k*}.

**Definition 11**

Let $\bar{x}\in X$. We say that $\bar{x}$ is a weak efficient solution of ((IVP)) if there exists no $\tilde{x}\in X$ such that $f(\tilde{x})≺f(\bar{x})$.

## 3. Necessary Optimality Conditions

In this section, we establish Fritz John and Karush-Kuhn-Tucker necessary optimality conditions for problem ((IVP)).

**Theorem 12 (Fritz John necessary condition)**

Assume that x* is an efficient solution for ((IVP)). If  (*df* ^*L*^)^+^(*H*_x*,*x*_(0^+^)), (*df* ^*U*^)^+^(*H*_x*,*x*_(0^+^)), and (*dg*_*J*(x*)_)^+^ (*H*_x*,*x*_(0^+^)) are convex-like on *X* with respect to the variable *x* and  *g*_*j*_ is upper semicontinuous at x* for *j* ∈ *J*∖*J*(x*), then there exist *ξ* ^*L*^,   *ξ* ^*U*^ ∈ ℝ_+_^*k*^, *μ* ∈ ℝ_+_^*m*^, (*ξ* ^*L*^, *ξ* ^*U*^, *μ*) ≠ 0 such that

(9) (ξL)T(dfL)+(Hx∗,x(0+))+(ξU)T(dfU)+(Hx∗,x(0+))+μT(dg)+(Hx∗,x(0+))≧0, ∀x∈X;

(10) $$\begin{array}{c} \mu^{T}g\left(x^{*}\right)=0. \end{array}$$

**Proof**

We prove firstly that the following system of inequalities

(11) $$\begin{array}{c} {\left(df^{\alpha }\right)}^{+}\left(H_{x^{*},x}\left(0^{+}\right)\right)<0,\;s\in \left\{L,U\right\};\\ {\left(\text{d}g_{J(x^{*})}\right)}^{+}\left(H_{x^{*},x}\left(0^{+}\right)\right)<0 \end{array}$$

has no solution for *x* ∈ *X*. We proceed by contradicting. If there exists *x*′ ∈ *X*, a solution of this system, we get that for each $i=\bar{1,n}$ there exist *δ*_*i*_^*L*^ > 0 and *δ*_*i*_^*U*^ > 0 such that for *s* ∈ {*L*, *U*}

(12) $$\begin{array}{c} f_{i}^{s}\left(H_{x^{*},x^{\prime }}\left(t\right)\right)<f_{i}^{s}\left(x^{*}\right),\;\text{for}\;t\in \left(0,\delta_{i}^{s}\right), \end{array}$$

and for each *j* ∈ *J*(x*) there exists *δ*_*j*_ > 0 such that

(13) $$\begin{array}{c} g_{j}\left(H_{x^{*},x^{\prime }}\left(t\right)\right)<g_{j}\left(x^{*}\right)=0,\;\text{for}\;t\in \left(0,\delta_{j}\right). \end{array}$$

Since *g*_*j*_, for *j* ∉ *J*(x*), is semicontinuous at x*, then *g*_*j*_(*H*_x*,*x*′_(*t*)) is semicontinuous at *t* = 0. Finally we get

(14) $$\begin{array}{c} f_{i}^{s}\left(H_{x^{*},\bar{x}}\left(t\right)\right)<f_{i}^{s}\left(x^{*}\right),\;\text{for}\;i\in K,\;s\in \left\{L,U\right\};\\ g_{j}\left(H_{x^{*},x^{\prime }}\left(t\right)\right)<0,\;\text{for}\;j\in M, \end{array}$$

for any *t* ∈ (0, *δ*_0_), where *δ*_0_ = min⁡{min⁡_*i*_ *δ*_*i*_^*L*^, min⁡_*i*_ *δ*_*i*_^*U*^, min⁡_*j*_ *δ*_*j*_} > 0. These inequalities contradict that x* is a weak efficient solution for ((IVP)). Hence the systems (9) and (10) have no solution for *x* ∈ *X*. Now we can apply Lemma 8. Since (*df* ^*L*^)^+^, (*df* ^*U*^)^+^, and (*dg*_*J*(x*)_)^+^(*H*_x*,*x*_(0^+^)) are convex-like on *X*, we obtain that (9) and (10) hold and the proof is complete.

**Theorem 13 (Karush-Kuhn-Tucker necessary optimality condition)**

Let (*df* ^*L*^)^+^(*H*_x*,*x*_(0^+^)), (*df* ^*U*^)^+^(*H*_x*,*x*_(0^+^)), and (*dg*_*J*(x*)_)^+^(*H*_x*,*x*_(0^+^)) be convex-like on *X* with respect to the variables *x* and *g*_*j*_, for *j* ∉ *J*(x*), be upper semicontinuous at x*. If there exists *x*′ ∈ *X*_0_ such that

(15) $$\begin{array}{c} {\left(dg_{J(x^{*})}\right)}^{+}\left(H_{x^{*},x^{\prime }}\left(0^{+}\right)\right)<0, \end{array}$$

and x* is a weak efficient solution for the problem ((IVP)), then there exist *ξ* ^*α*^ = (*ξ*_1_^*α*^,…,*ξ*_*k*_^*α*^)^*T*^ ∈ ℝ_+_^*k*^, *α* ∈ {*L*, *U*}, and *μ* ∈ ℝ_+_^*m*^ satisfying (9), (10), and (*ξ* ^*L*^, *ξ* ^*U*^) ≠ 0.

**Proof**

We suppose (*ξ* ^*L*^, *ξ* ^*U*^) = 0. Then, by (9) we obtain

(16) $$\begin{array}{c} \mu^{T}{\left(dg\right)}^{+}\left(H_{x^{*},x}\left(0^{+}\right)\right)≧0,\;\forall x\in X. \end{array}$$

Since *μ*_*j*_ = 0 for *j* ∈ *J*∖*J*(x*), by (10), and there exists *j* ∈ *J*(x*) such that *μ*_*j*_ > 0, by (15), it results *μ* ^*T*^(*dg*)^+^(*H*_x*,*x*′_(0^+^)) < 0, which contradicts (15) and theorem is proved.

## 4. Sufficient Optimality Conditions

In this section we give some Karush-Kuhn-Tucker type sufficient optimality conditions under generalized convexity with upper-Dini-derivative concept.

**Theorem 14**

Let x* be a feasible solution of ((IVP)). Assume that there exist *ξ* ^L*^, *ξ* ^U*^, (*ξ* ^L*^, *ξ* ^U*^) ≠ 0 and μ*≧0, *i* ∈ *J*(x*), such that *f*_*i*_^*s*^  (*i* ∈ *K*, *s* ∈ {*L*, *U*}) and *g*_*j*_  (*j* ∈ *J*) are (*α*, *ρ*_*i*_^*s*^) and (*α*, *ρ*_*j*_^  ′^)-right upper-Dini-derivative locally arcwise connected at x* with respect to *H*, respectively. Also one assumes *α*(*x*, x*)≧0 and

(17) $$\begin{array}{c} {\left(\xi^{L^{*}}\right)}^{T}\rho^{L}\left(x,x^{*}\right)+{\left(\xi^{U^{*}}\right)}^{T}\rho^{U}\left(x,x^{*}\right)+\mu_{J\left(x^{*}\right)}^{*T}\rho_{J\left(x^{*}\right)}^{{\;}^{\prime }}≧0 \end{array}$$

hold for all *x* ∈ *X*_0_ feasible. Then x* is a weak efficient solution for ((IVP)).

**Proof**

We suppose to the contrary that x* is not a weak efficient solution for ((IVP)). Then there exists *x*′ ∈ *D* such that *f*(*x*′)≺*f*(x*). Now, since (*ξ* ^L*^, *ξ* ^U*^) ≥ 0, *μ*_*J*(x*)_*≧0, and *g*_*J*(x*)_(*x*′)≦0 = *g*_*J*(x*)_(x*), we get

(18) (ξL∗)T(fL(x′)−fL(x∗))+(ξU∗)T(fU(x′)−fU(x∗))+μJ(x∗)  ∗T(gJ(x∗)(x′)−gJ(x∗)(x∗))<0.

Since *f*_*i*_^*s*^  (*i* ∈ *K*, *s* ∈ {*L*, *U*}) and *g*_*j*_  (*j* ∈ *J*) are (*α*, *ρ*_*i*_^*s*^) and (*α*, *ρ*_*j*_^  ′^)-right upper-Dini-derivative locally arcwise connected at x*, by (18) we obtain

(19) α(x′,x∗)((ξL∗)T(dfL)+(Hx∗,x′(0+))) +(ξU∗)T(dfU)+(Hx∗,x′(0+)) +μJ(x∗)∗T(dgJ(x∗))+(Hx∗,x′(0+)) +(ξL∗)TρL(x¯,x′)+(ξU∗)TρU(x¯,x′)<0.

Now, by $\alpha (\bar{x},x^{\prime })≧0$ and (17) we get a contradiction. Thus the theorem is proved.

The next sufficient optimality condition is given in the case of generalized pseudo- and quasi-right upper-Dini-derivative arcwise connected type, where the proof is on the line of the above theorem.

**Theorem 15**

Let x* be a feasible solution of ((IVP)). Assume that there exist *ξ* ^L*^, *ξ* ^*U*∗^, (*ξ* ^L*^, *ξ* ^U*^) ≥ 0 and μ*≧0, *i* ∈ *J*(x*), such that *f* ^*s*^  (*s* ∈ {*L*, *U*}) is generalized pseudoright upper-Dini-derivative locally arcwise connected at x* with respect to *H* and *g* is *ρ*′-generalized quasi-right upper-Dini-derivative locally arcwise connected at x* with respect to *H*, respectively. Also one assumes that there exist *ξ* ^L*^, *ξ* ^U*^, (*ξ* ^L*^, *ξ* ^U*^) ≥ 0 and μ* ≥ 0 such that (*ξ* ^L*^)^*T*^ *ρ* ^*L*^(*x*, x*) + (*ξ* ^U*^)^*T*^ *ρ* ^*U*^(*x*, x*) + *μ* ^∗*T*^ *ρ* ^′^(*x*, x*)≧0 for any *x* ∈ *X*_0_. Then x* is a weak efficient solution for ((IVP)).

## 5. Wolfe Duality

Relative to ((IVP)) we consider the following Wolfe type dual problem:

(IWVD)min⁡  Ψ(y,ζL,ξU,μ)=f(y)+μTg(y)esubject  to  (ξL∗)T(dfL)+(Hy,x(0+))      +(ξU∗)T(dfU)+(Hy,x(0+))      +μT(dg)+(Hy,x(0+))≧0, ∀x∈X0      (ξL,ξU)≥0,  μ≧0, y∈X,

where *f*(*y*) = [*f* ^*L*^(*y*), *f* ^*U*^(*y*)] and *e* = (1,1,…, 1) such that [*e* ^*T*^ *ξ* ^*L*^, *e* ^*T*^ *ξ* ^*U*^] = [1,1] = 1. Let *F*_*D*_ denote the set of all feasible solutions of ((IWVD)) and let pr_*X*_ *F*_*D*_ be the projection of the set *F*_*D*_ on *X*. Now we present weak, strong, and strict converse duality theorems relative to ((IVP)) and ((IWVD)). The proofs, which will be skipped here, follow the classic lines of multiobjective optimization [4, 7] and interval optimization problems [11].

**Theorem 16 (weak duality)**

Let *x* and (*y*, *ξ* ^*L*^, *ξ* ^*U*^, *μ*) be a feasible solution for ((IVP)) and ((IWVD)), respectively. One supposes that *f* ^*s*^  (*s* ∈ {*L*, *U*}) and *g* are (*α*, *ρ* ^*s*^) and (*α*, *ρ*′)-right upper-Dini-derivative arcwise connected at *y* on *X*_0_ ∪ pr_*X*_ *F*_*D*_ with respect to *H*, respectively. Moreover, one assumes *α*(*x*, *y*)≧0 and (*ξ* ^*L*^)^*T*^ *ρ* ^*L*^ + (*ξ* ^*U*^)^*T*^ *ρ* ^*U*^ + *μ* ^*T*^ *ρ*′≧0, for all *x* ∈ *X*_0_. Then the following cannot hold: *f*(*x*)≺Ψ(*y*, *ξ* ^*L*^, *ξ* ^*U*^, *μ*).

**Theorem 17 (strong duality)**

Let x* be a weak efficient solution of ((IVP)), at which the assumptions of Karush-Kuhn-Tucker necessary optimality conditions are satisfied. Then there exist (*ξ* ^L*^, *ξ* ^U*^) ≥ 0 and μ* ∈ ℝ^*m*^ such that (x*, *ξ* ^L*^, *ξ* ^U*^, μ*) ∈ *F*_*D*_ and the objective values of ((IVP)) and ((IWVD)) are equal. Further, if the hypotheses of weak duality theorem hold for all feasible solutions (*y*, *ξ* ^*L*^, *ξ* ^*U*^, *μ*) for ((IWVD)), then (x*, *ξ* ^L*^, *ξ* ^U*^, μ*) is an efficient solution of ((IWVD)).

**Theorem 18 (converse duality)**

Let (y*, *ξ* ^L*^, *ξ* ^U*^, μ*) be a weak efficient solution of ((IWVD)). One assumes that *f* ^*s*^  (*s* ∈ {*L*, *U*}) and *g* are (*α*, *ρ* ^*s*^) and (*α*, *ρ*′)-right upper Dini derivative arcwise connected at *y* on *X*_0_ ∪ pr_*X*_ *F*_*D*_ with respect to *H*, respectively. If *α*(*x*, y*)≧0 and (*ξ* ^L*^)^*T*^ *ρ* ^*L*^(*x*, y*) + (*ξ* ^U*^)^*T*^ *ρ* ^*U*^(*x*, y*) + (μ*)^*T*^ *ρ*′(*x*, y*)≧0, for all *x* ∈ *X*_0_, then y* is a weak efficient solution of ((IVP)).

## 6. Mond-Weir Duality

In this section we consider the following interval multiobjective dual problem, which is Mond-Weir dual type [6]:

(IMWVD)max⁡  f(y)subject  to    (ξL)T(dfL)+(Hy,x(0+))     +(ξU)T(dfU)+(Hy,x(0+))     +μT(dg)+(Hy,x(0+))≧0, ∀x∈X0     μTg(y)≧0     ξL,ξU∈ℝn, (ξL,ξU)≥0, μ≧0,

where *e* = (1,…, 1) ∈ ℝ^*k*^, with [*e* ^*T*^ *ξ* ^*L*^, *e* ^*T*^ *ξ* ^*U*^] = [1,1] = 1. Let *F*_*D*_ denote the set of all feasible solutions of ((IMWVD)) and pr_*X*_ *F*_*D*_ as the projection of the set *F*_*D*_ on *X*.

As in Section 5, we can establish some duality results. Here, we simply state them in the next theorems.

**Theorem 19 (weak duality)**

Let *x* and (*y*, *ξ* ^*L*^, *ξ* ^*U*^, *μ*) be a feasible solution for ((IVP)) and ((IMWVD)), respectively. Assume that *f* ^*s*^(*s* ∈ {*L*, *U*}) and *g* are (*α*, *ρ* ^*s*^) and (*α*, *ρ*′)-right upper-Dini-derivative arcwise connected at *y* on *X*_0_ ∪ pr_*X*_ *F*_*D*_ with respect to *H*, respectively. Also one supposes that *α*(*x*, *y*)≧0 and (*ξ* ^*L*^)^*T*^ *ρ* ^*L*^(*x*, *y*) + (*ξ* ^*U*^)^*T*^ *ρ* ^*U*^(*x*, *y*) + *μ* ^*T*^ *ρ*′(*x*, *y*)≧0, for all *x* ∈ *X*_0_. Then the following cannot hold: *f*(*x*)⪯*f*(*y*).

**Theorem 20 (strong duality)**

Let x* be a weak efficient solution of the interval multiobjective programming problem ((IVP)), at which the assumptions of Karush-Kuhn-Tucker necessary optimality conditions are satisfied. Then there exist *ξ* ^L*^, *ξ* ^U*^ ∈ ℝ^*k*^, (*ξ* ^L*^, *ξ* ^U*^) ≥ 0, and μ* ∈ ℝ^*m*^, μ*≧0, such that (x*, *ξ* ^L*^, *ξ* ^U*^, μ*) is a feasible solution for (*IMWVD*) and the objective values of ((IVP)) and ((IMWVD)) are equal. Moreover, if the weak duality result between ((IVP)) and ((IMWVD)) holds, then (x*, *ξ* ^L*^, *ξ* ^U*^, μ*) is a weak efficient solution for ((IMWVD)).

**Theorem 21 (converse duality)**

Let (y*, *ξ* ^L*^, *ξ* ^U*^, μ*) be a weak efficient solution of ((IMWVD)). Suppose that *f* ^*s*^  (*s* ∈ {*L*, *U*}) and *g* are (*α*, *ρ* ^*s*^) and (*α*, *ρ*′)-right upper-Dini-derivative arcwise connected at *y* on *X*_0_ ∪ pr_*X*_ *F*_*D*_ with respect to *H*, respectively. Further, if *α*(*x*, y*)≧0 and (*ξ* ^L*^)^*T*^ *ρ* ^*L*^(*x*, y*)+(*ξ* ^U*^)^*T*^ *ρ* ^*U*^(*x*, y*)+(μ*)^*T*^ *ρ*′(*x*, y*)≧0, for all *x* ∈ *X*_0_, then y* is a weak efficient solution of ((IVP)).

## 7. Conclusions

In this paper we considered an interval multiobjective optimization problem. Some new optimality conditions and duality results were studied under the generalized convexity considered by Yuan and Liu [1]. Necessary optimality conditions and sufficient optimality conditions were derived. Duality results were established. These results can be extended to a class of univex generalized convexity of Mishra type [4] with the tool-right upper-Dini-derivative. Further it is possible to establish duality results relative to a mixed dual of Xu type [9].
